# Exploring the effect of different typical plant community on human stress reduction: a field experiment

**DOI:** 10.1038/s41598-024-56243-7

**Published:** 2024-03-07

**Authors:** Wenfei Yao, Qingzi Luo, Xiaofeng Zhang, Chen Zhuo, Longfei Mi

**Affiliations:** 1 College of Architecture and Urban Planning, Qingdao University of Technology, Qingdao, Shandong People’s Republic of China; 2 College of Landscape Architecture, Nanjing Forestry University, Nanjing, Jiangsu People’s Republic of China; 3 Engineering Research Center of Concrete Technology Under Marine Environment, Ministry of Education, Qingdao University of Technology, Qingdao, China

**Keywords:** Stress reduction, Plant community, Natural exposure, Psychological effects, Physiological effects, Psychology, Psychology and behaviour

## Abstract

Research has demonstrated the positive effect of natural environment on human restoration and well-being. Time spent in nature can often alleviate both physiological and psychological stress. However, few studies have discussed the environmental health effects of the nature’s components and characteristics. Sixty volunteers were recruited and one manufactured environment and five different natural environments were randomly assigned to them, including coniferous forests (pure coniferous forest-PC and mixed coniferous forest-MC), broad-leaved forests (pure broad-leaved forest-PB and mixed broad-leaved forest-MB), and mixed forest (mixed coniferous and broad-leaved forest-MCB). Each volunteer sat in a built or natural environment and looked around the environment for 15 min. Physiological (HR, HRV, BP, pulse rate and salivary cortisol) and psychological indicators (POMS and STAI) were used to evaluate the changes in their stress level. Results indicated a strong difference in HR, HRV, POMS and STAI between the built and natural environment, which showed that natural environment can lower the stress level. MC had the best effect on relieving physiological stress, whereas MCB is most successful in improving emotional state and reducing anxiety. Broad-leaved forest and mixed forest significantly affected the DBP and vigor level of the subjects, respectively. While coniferous forest did significantly increase the concentration of salivary cortisol in subjects. The study confirmed that compared to the built environment, the natural environment can relieve the human body's physical and psychological stress and negative emotions, while significantly increasing vitality. And different plant communities also have different effects on the physiological and psychological indicators of the subjects. These results will provide scientific basis for the construction and improvement of urban green space environment.

## Introduction

Since the beginning of the twenty-first century, there has been an increasing trend of global urbanization. At present, more than half of the world’s population lives in cities, making urbanization one of the main health-related issues we are currently facing and will continue to face in the future^1,2^. Vlahov and Galea^3^ mentioned that city health had frequently mirrored the movement and growth of the population shaping the urban landscape. The increasingly close relationship between human well-being and ecosystem health links urban development to population health^4^. However, people living in cities tend to have higher probability of mental illness, which aggravates people’s concern about health problems^5^. The stress caused by urbanization has become a public health problem, which seriously affects the healthy development of individuals, groups and societies.

Natural environment is increasingly regarded as vital to both physical and mental health and subjective well-being and there may be a positive correlation between natural exposure and cognitive and behavioral development^6,7^. The widely recognized attention recovery theory (ART) and stress recovery theory (SRT) explain the natural environment’s effect on human health and they support the view that natural environment has become an important barrier between healthy and unhealthy lifestyles of human beings^8^. Contact with urban green space can help the psychological and physiological states of the human body, as well as reduce stress and attention fatigue^9^. Previous research has shown that short-term exposure to nature is associated with a more positive mental and physical state^10–12^. Exposure to the nature plays a positive role in promoting human health and well-being, as evidenced by the effective relief of various physical diseases^13–15^, the improvement of emotional state^16,17^, and the promotion of a healthy and active lifestyle etc*.*^18,19^. At the moment, the majority of studies compare people’s health before and after exposure to greenery. Such experimental studies have been repeated with various participant characteristics (age, health state, sex, etc.)^20–23^, different intervention times (ranging from minutes to months)^24,25^ and different environment types (forest, urban park, blue space, even virtual green space, etc.)^13,26–28^. This fully demonstrates the universality and equity of the impact of the natural environment on human health, which plays an important role in guiding social equity and enhancing the resilience of individuals and societies.

Hoyle et al.^29^ proposed that there was a complex relationship between the effects of people’s perception of aesthetics, recovery and well-being and environmental biodiversity, all of which were related to the optimization and management of urban green spaces. Some previous research has focused on the variations of levels of health in humans caused by different natural environments. Chiang et al.^30^ investigated the effects of different geographical locations and the vegetation density on human health. The findings revealed that different landscapes had distinctive effects on stress, attention restoration, and positive emotions, etc. An experiment conducted in forests with different tree species confirmed that there are some health differences caused by varying natural environments^30^. Furthermore, the same differences were also noticed in the experiments carried out near diverse roadside tree species^31^. These variations were attributed to the change of the physical environment caused by natural environment properties. In addition, the level of greenery and the degree of human intervention both have different impacts on human health^31,32^. Takayama^32^, for example, concluded that managed forests were more beneficial to the emotional well-being than wild forests. A study by Liu showed that spatial naturalness and composition factors greatly affect the restorative quality of the environment^33^. In addition, some indoor experiments involved the multi-sensory effects of human body, such as sounds^34^, smells^35^ or the combination of the two^36^. These results support the conclusion that people have different responses to different natural environments, and also promote the integration of urban development, green space equity, and human exposure^37^.

However, the majority of existing outdoor studies have been carried out in urban forests or parks, and there has been little research on smaller-scale plant communities. Plant communities, as fundamental components of urban green spaces, play an important role in landscape architecture, particularly in the creation of urban microclimates. Therefore, studying the differences in the influence of different plant communities on human healthy has become an important strategy for creating a healthy living environment. In this study, five different natural environments were selected, including coniferous forests (n = 2), broad-leaved forests (n = 2), and mixed forest (n = 1). Because the complex psycho-physiological pathways of stress make single marker measurements impossible, a subjective psychological scale and multiple physiological indicators related to physiological stress response were used to record changes in stress levels. Based on the information provided above, we assumed that people exposed to the natural environment will experience less stress than those exposed to the built environment, and that different plant community spaces will have diverse effects on human stress indicators. Therefore, the aim of the study was to determine whether the natural environment has a positive effect on human stress reduction and to explore whether this positive effect varies by plant community type.

## Materials and methods

### Experimental sites

**Figure 1:**
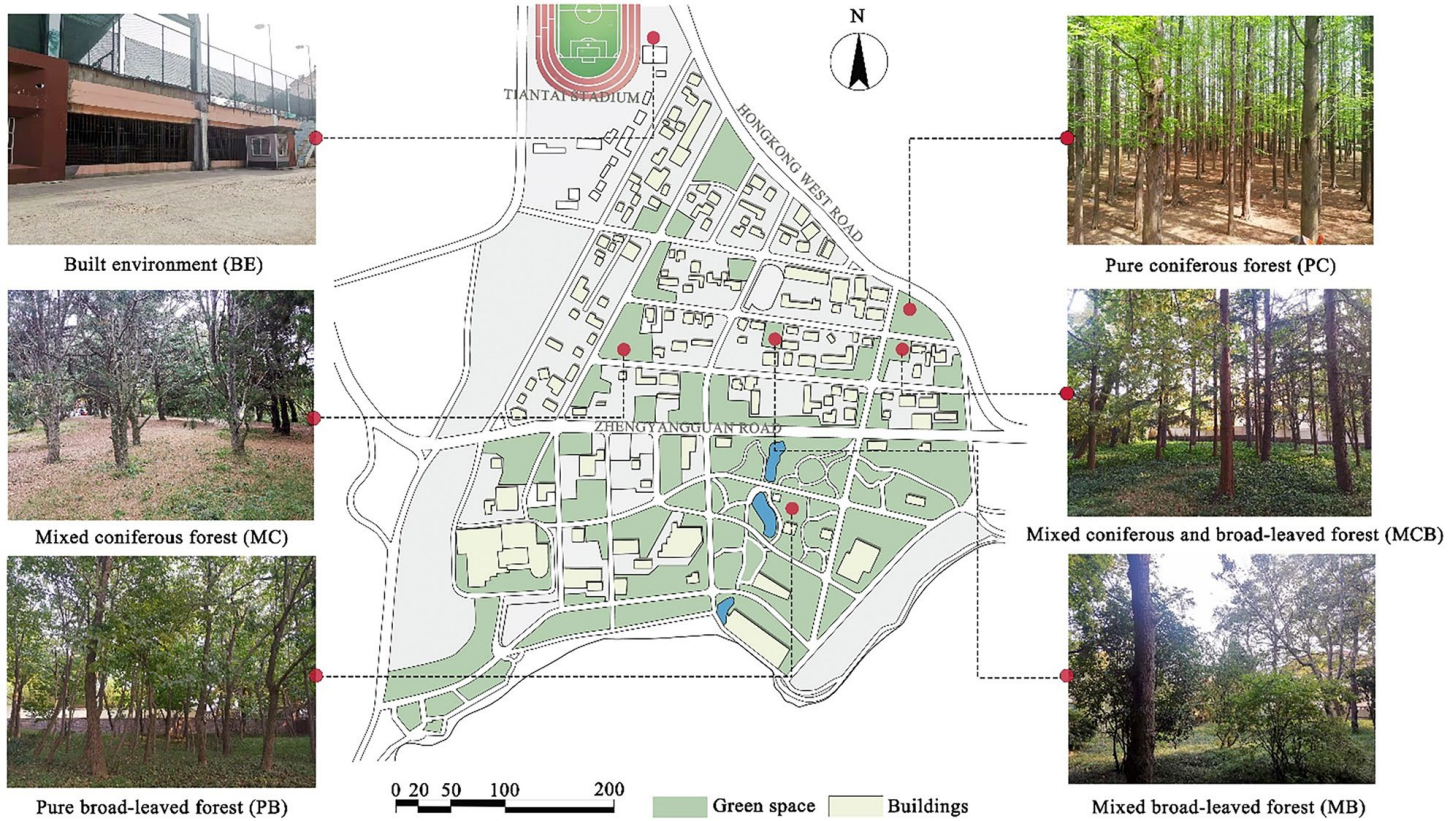
Experimental sites.

This research was conducted in the Badaguan Scenic Area, located in Qingdao, Shangdong Province. It covers an area of approximately 70 hectares and is a well-known scenic health resort in China. The location was chosen for its lush vegetation and excellent natural conditions. To determine the impact of different plant communities on stress levels, five 20 × 20 m (400 m^2^) plots of plant communities were selected in different plant composition and spatial structure for research, including coniferous forests (pure coniferous forest-PC and mixed coniferous forest-MC), broad-leaved forests (pure broad-leaved forest-PB and mixed broad-leaved forest-MB), and mixed forest (mixed coniferous and broad-leaved forest-MCB) (Fig. 1). A gymnasium near the Badaguan Scenic Area was chosen as a built environment (BE). The built environment is devoid of greenery and is surrounded by buildings, football pitches and tennis courts.

### Environments data

The climate of Qingdao is very suitable, with an average yearly temperature of 12.7 °C. This research was conducted from May to June of 2021. The environmental characteristics of plant communities were collected in September and October 2021. The climate in these two periods was similar. The values were measured every day from 8:00 to 18:00 and the hourly average was used to indicate each day’s variations. The average of all the data is used to describe the environmental characteristics of each plot.

As shown in Table 1, the temperature of natural environment was lower than that of the built environment, whereas the relative humidity was higher, confirming the cooling and humidification properties of the natural environment. MCB had the lowest temperature (26.39 ± 0.66 °C), while MB had the highest relative humidity (74.68 ± 3.30). There was no significant difference in particulate matter concentration (PM_2.5_, PM_10_) between the environments. The plant community with the lowest particulate matter concentration was MB (PM_2.5_: 31.54 ± 1.75 μg/m^3^; PM_10_: 34.88 ± 2.95 μg/m^3^), while the highest was MC (PM_2.5_: 84.71 ± 11.97 μg/m^3^; PM_10_: 63.33 ± 6.10 μg/m^3^).

**Table 1:** Details of experiment sites.

Items	Coniferous forest		Broad-leaved forest		Mixed forest	Control group
	PC	MC	PB	MB	MCB	BE
Chief species	Metasequoia glyptostroboides	Juniperus formosana; Pinus bungeana; Pinus armandii; Cedrus deodara;	Eucommia ulmoides	Paulownia fortune; Quercus dentata; Pterocarya stenoptera; Photinia serrulata; Forsythia suspensa;	Cedrus deodara; Metasequoia glyptostroboides; Robinia pseudoacacia; Prunus cerasifera;	–
Temperature (°C) (mean ± SD)	26.53 ± 1.05	27.36 ± 1.01	27.33 ± 1.19	27.50 ± 0.76	26.39 ± 0.66	29.03 ± 1.30
Humidity (%) (mean ± SD)	67.64 ± 5.59	70.60 ± 3.22	69.47 ± 3.29	74.68 ± 3.30	69.98 ± 3.35	54.84 ± 6.93
PM_2.5_ (μg/m^3^) (mean ± SD)	52.75 ± 20.08	84.71 ± 11.97	54.25 ± 7.72	31.54 ± 1.75	41.00 ± 4.10	33.76 ± 8.12
PM_10_ (μg/m^3^) (mean ± SD)	61.43 ± 19.58	89.94 ± 12.44	63.33 ± 6.10	34.88 ± 2.95	49.59 ± 6.63	39.42 ± 10.66

*PC* pure coniferous forest, *MC* mixed coniferous forest, *PB* pure broad-leaved forest, *MB* mixed broad-leaved forest, *MCB* mixed coniferous and broad-leaved forest, *BE* built environment.

### Participants

Through poster posting and offline promotion, 60 undergraduate and graduate students aged 18 to 25 were recruited (Table 2). All of the subjects were healthy and had no previous history of cardiovascular, salivary or psychiatric disorders. All the participants volunteered to take part in the experiment and they would spend time in every plant community and the built environment. In the end, a total of 59 subjects participated in the experiment (Mean ± SD: 21.17 ± 2.19; Male = 18; Female = 41). Mean height and weight were 167.07 ± 7.43 cm and 58.64 ± 8.72 kg. The body mass index (BMI) was 20.89 ± 2.32 kg/m^2^. There was no statistically significant difference in baseline data between the groups. The study was conducted in compliance with the WMA Declaration of Helsinki and was performed with the approval of the local Ethics Committee of Qingdao University of Technology, China. All the participants were informed about the research’s purpose, process and signed an informed consent form before the experiment.

**Table 2:** Participant Details (N = 59).

Items	PC	MC	PB	MB	MCB	BE	Total
Number	10	10	10	10	9	10	59
Age (years)	21.00 ± 1.81 (19.63, 22.37)	22.29 ± 2.00 (20.78, 23.80)	19.98 ± 1.08 (19.17, 20.79)	20.61 ± 1.90 (19.18, 22.04)	21.17 ± 2.47 (19.16, 23.18)	21.99 ± 2.68 (19.97, 24.01)	21.17 ± 2.19 (20.60, 21.75)
Sex	Male = 2; Female = 8;	Male = 3; Female = 7;	Male = 3; Female = 7;	Male = 3; Female = 7;	Male = 2; Female = 7;	Male = 5; Female = 5	Male = 18; Female = 41;
Height (cm)	168.90 ± 7.56 (163.20, 174.60)	165.60 ± 6.51 (160.69, 170.51)	165.90 ± 4.41 (162.57, 169.23)	171.80 ± 8.47 (165.41, 178.19)	164.00 ± 8.35 (157.19, 170.81)	165.90 ± 5.75 (161.56, 170.24)	167.07 ± 7.43 (165.11, 169.02)
Weight (kg)	59.40 ± 6.53 (54.48, 64.32)	57.00 ± 10.03 (49.44, 64.56)	59.70 ± 7.83 (53.79, 65.61)	59.50 ± 11.53 (50.81, 68.19)	55.00 ± 8.59 (47.98, 62.00)	59.80 ± 5.13 (55.93, 63.67)	58.64 ± 8.72 (56.17, 60.75)
BMI (kg/m^2^)	20.84 ± 2.13 (19.24, 22.45)	20.68 ± 2.79 (18.58, 22.78)	21.68 ± 2.70 (19.64, 23.72)	19.98 ± 1.88 (18.56, 21.40)	20.36 ± 2.03 (18.70, 22.02)	21.73 ± 1.52 (20.58, 22.87)	20.89 ± 2.32 (20.28, 21.50)

*BMI* body mass index, *STAI* state-trait anxiety scale.

The value of 95% confidence interval (95% CIs) follows the mean and standard deviation closely.

### Physiological measurement

#### Blood pressure and pulse rate

A digital blood pressure monitor (HEM-8713, Omron, China) was used to measure and record blood pressure (systolic and diastolic blood pressure, SBP and DBP) and pulse rate. The measurement was taken on the upper arm of the subject’s non-dominant arm. Both measurements were taken in the same manner before and after the experiment while the subject was sitting. SBP, DBP and pulse rate were directly recorded both before and after experiment, such as SBP = 132 bpm before the experiment and SBP = 125 bpm after the experiment.

#### HR and HRV

Heart rate (HR) and heart rate variability (HRV) were measured via a Polar heart rate chest belt (Polar H10) and a wristwatch (Polar Unite). This is a wearable biofeedback sensor, which has been widely used in similar experiments^38,39^. The heart rate sensor was placed on the belt around the chest, close to the chest cavity. Additionally, the subjects wore a wristwatch on their left hand. The heart rate belt used the micro-voltage signals (ECG) generated by the heartbeat to directly amplify and process the subjects’ heart rate value. Furthermore, the heart rate belt and wristwatch also recorded RR intervals data which was used for HRV analysis. The cubic spline interpolation method was used for artifact correction and trend removal of RR intervals^40^. The corrected RR intervals were logged into the Kubios HRV Standard 3.5.0, which was more suitable for HRV analysis in large animals or humans^41^.

Both time and frequency domains of HRV were calculated according to the RR intervals. The HF and LF/HF ratio in the frequency domain were used to represent the status of the subjects’ autonomic neural activity. Among them, HF serves as the indicator of parasympathetic nerve activity, increasing in a relaxed state. The LF/HF ratio reflects the relative activity of both sympathetic and parasympathetic nerves^42,43^. A higher HF value and a lower LF/HF ratio represent a more relaxed state. To normalize the HRV parameters in the analysis, natural logarithmic transformed values were used^44^.

#### Salivary cortisol

Salivary cortisol is a reliable and valid parameter of human stress response^45,46^. Salivary cortisol samples were collected in a salivette (No. 51.1534.500; Sarstedt, Numbrecht, Germany), ensuring a safe and non-invasive collection process. Saliva samples were collected both before and after the experiment to compare the responses to the environmental stimuli (for example pre = 1.88 ug/L and pos = 1.75 ug/L). The collected saliva samples were immediately frozen and sent to a laboratory (Qingdao, China) for Enzyme-Linked ImmunoSorbent Assay (ELISA) determination.

### Psychological measurement

#### POMS

Profile of mood states (POMS) is a reliable measure of momentary mood state, which has been previously used to evaluate the effect the natural environment has on the emotions^31,47,48^. This study adopted a short form of POMS with 30 items as a means of evaluation of the subjects’ emotional state^13^. This short scale retained the six dimensions of human emotion evaluation: tension-anxiety (T-A), depression (D), anger-hostility (A-H), vigor (V), fatigue (F) and confusion (C). Each question was rated on a five-point Likert scale from 0 (almost none) to 4 (very much), while each subscale had a definite score. The value named “Total Mood Disturbance (TMD)” was used to describe the total emotional state of the human body, which was calculated by [(T-A + D + A-H + F + C)—V]^13^. In the experimental samples of this study, the overall internal consistency of the POMS scale was shown as Cronbach's α = 0.84.

#### STAI

To compare the changes in the participants’ anxiety levels in each plant community, a 40-item State Trait Anxiety Scale (STAI), including the Trait Anxiety Inventory (TAI) and State Anxiety Inventory (SAI), was used to ask how anxious the participants felt^49^. SAI is meant to measure the level of anxiety in the present moment and TAI is meant to measure anxiety levels as a personal characteristic. Subjects were asked to use a four-point Likert scale to express their feelings regarding anxiety ranked from 1 (almost never) to 4 (always). The question score of both subscales would be used to describe the participants’ state anxiety and trait anxiety levels. In the original Spanish validation, STAI-state showed an internal consistency of Cronbach’s α = 0.90, and in the current study, good internal consistency of STAI-T with Cronbach’s α = 0.86, and STAI-S with Cronbach’s α = 0.78 was detected^50^.

### Procedure

**Figure 2:**
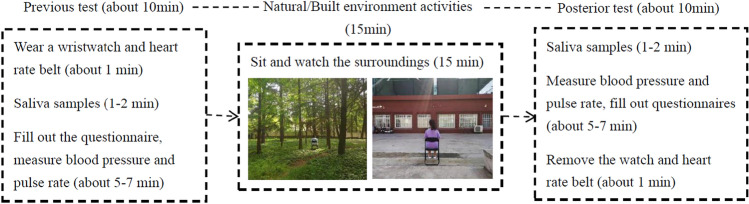
Experimental process.

This study employed a randomized parallel experiment. All 60 volunteers were randomly divided into six groups of ten. Each group of volunteers was randomly matched to either a natural or built environment and completed a pre-post test and a 15-min meditation session. The physiological and psychological conditions were measured 10 min after the participant arrival at the site. Meanwhile, they were informed of the experimental procedure and important matters. Before the experiment, all the subjects were given heart rate belts and wristwatches which were secured by the researchers. Subsequently, their salivary cortisol was measured, as well as their blood pressure and pulse rate. Additionally, they filled out the psychological evaluations. Once this was done, each participant would sit in a chair placed in the center of the field for 15 min. Cell phones and talking were not allowed during the experiment. After the exposure, subjects walked out of the field and all of the measurements were taken again. Finally, the researcher ended the experiment by uploading the record from the wristwatch. The whole procedure would last for approximately 30 to 40 min. The specific process is shown in Fig. 2.

### Data analysis

A total of 60 participants were recruited for the study. During the experiment, one person was absent due to personal reasons, and two people failed to record heart rate and heart rate variability due to faulty instruments. The changes of psychological scales, blood pressure, pulse rate, and salivary cortisol concentrations were compared directly before and after the experiment. To compare differences in HR and HRV, 15-min and one-minute mean values were used. Data results were analyzed in Microsoft Office Excel and SPSS 22.0. We test the normality of the dependent variable through visual plots (histograms and normal probability plots) and Shapiro–Wilk, and then the homogeneity test of variance was performed. In order to determine whether there are differences in human stress indicators after 15 min of exposure to different environments, we conducted one-way ANOVA and multiple post-hoc comparisons (LSD) for variables satisfying homogeneity of variance, and non-parametric tests (Kruskal–Wallis) for variables not satisfying homogeneity of variance. A *p*-value less than 0.05 was taken as statistically significant.

### Approval for human experiments

The study was performed with the approval of the local Ethics Committee of the College of Qingdao University of Technology, China.

## Results

### Physiological effects of natural environment on human stress

#### Blood pressure and pulse rate

As shown in Tab.3, when compared with the built environment, the participants’ SBP did not significantly change after spending time in the five plant communities. The decline in DBP only appeared in PB (− 4.40 ± 3.69 mmHg) and BE (− 4.90 ± 5.79 mmHg). But compared with mixed forest, broad-leaved forest could significantly reduce DBP of subjects. After the 15-min exposure to both natural and built environment, the pulse rate of the participants had decreased, but the difference between groups was not significant. And the subjects’ pulse rate recorded in the built environment (76.22 ± 13.16 bpm) was higher than in the natural environment (69.27 ± 8.33 bpm). When it comes to the effects of the three plant communities, the pulse rates of subjects were all decreased. However, participants who were in PB (67.20 ± 5.60 bpm) had the lowest pulse rate after the experiment, while the pulse rate of the subjects who spent time in MC decreased the most (6.6 bpm), indicating that MC was the most effective when it comes to lowering the subjects’ pulse rate, followed by the MCB (4.78 bpm) and PB (3.20 bpm).

**Table 3:** Effects of plant community exposure on human blood pressure and pulse rate (N = 59).

Items	Data category	PC	MC	PB	MB	MCB	BE
SBP (mmHg)	PRE-test	111.00 ± 8.56 (104.55, 117.45)	105.60 ± 10.62 (97.59, 113.61)	115.70 ± 11.93 (106.70, 124.70)	105.50 ± 9.37 (98.43, 112.57)	107.56 ± 16.91 (93.77, 121.34)	108.90 ± 10.04 (101.33, 116.47)
	POS-test	111.40 ± 11.20 (101.31, 119.49)	104.00 ± 9.62 (96.74, 111.26)	112.80 ± 9.53 (105.62, 119.98)	105.30 ± 10.30 (97.54, 113.06)	108.22 ± 12.03 (97.26, 114.96)	103.90 ± 12.13 (94.75, 113.05)
DBP (mmHg)	PRE-test	71.60 ± 8.05 (65.53, 77.67)	65.50 ± 8.21 (59.31, 71.69)	77.90 ± 7.46 (72.65, 83.15)	69.50 ± 3.61 (66.78, 72.22)	68.89 ± 3.11 (66.36, 71.42)	71.40 ± 7.64 (65.64, 77.16)
	POS-test	74.50 ± 8.03 (68.45, 80.55)	66.50 ± 7.57 (60.79, 72.21)	73.50 ± 6.76 (68.41, 78.59)	70.60 ± 4.00 (67.58, 73.62)	73.78 ± 8.57 (66.79, 70.77)	66.50 ± 6.96 (61.25, 71.75)
Pulse rate (bpm)	PRE-test	73.30 ± 10.87 (65.10, 81.50)	75.20 ± 8.03 (69.14, 81.26)	70.40 ± 6.09 (65.81, 74.99)	71.30 ± 9.46 (65.07, 79.53)	75.00 ± 7.44 (68.93, 81.06)	77.78 ± 12.59 (64.02, 85.98)
	POS-test	71.80 ± 10.03 (64.35, 77.25)	68.60 ± 7.96 (62.59, 74.61)	67.20 ± 5.60 (62.98, 71.42)	69.60 ± 11.84 (60.67, 78.53)	70.22 ± 5.18 (66.00, 74.45)	76.22 ± 13.16 (62.47, 84.73)

#### Heart rate

**Figure 3:**
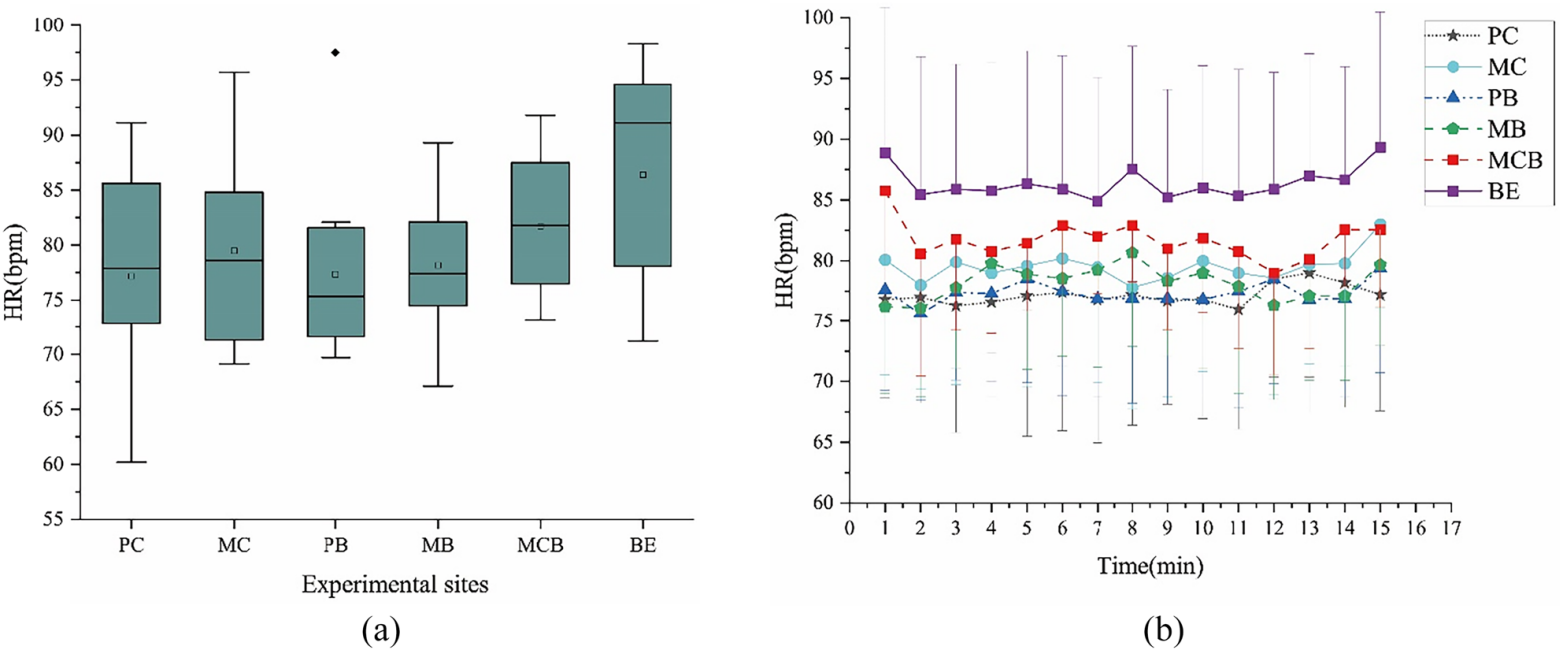
Mean 1-min HR and mean overall heart rate during viewing in natural and built environment. (**a**) Overall mean HR; (**b**) Changes in each 1-min average HR over the 15-min watch; N = 57.

There was a significant difference in HR between the 15-min exposure to the natural and built environment (F (1, 55) = 5.90, p = 0.018 < 0.05; 95%CIs-NE (natural environment) = 76.34, 81.15; 95%CIs-BE (built environment) = 78.17, 94.63) (Fig. 3), which was a set of data conforming to normal distribution and homogeneity of variance. After the 15-min exposure to the built environment, the average HR (86.40 ± 10.09 bpm) was significantly higher than that of the subjects who spent time in the natural environment (78.74 ± 8.20 bpm). The average HR of the subjects spending time in the PC was the lowest (77.17 ± 9.67 bpm), followed by those who spent time in the PB (77.36 ± 7.87 bpm) (Fig. 3a). In addition, subjects who were in the built environment had a higher mean HR within the 1-min segments than those in the natural environment. The average HR of the subjects exposed to the mixed forest (81.73 ± 6.13) was higher than that of the other two types of plant communities, which made the overall difference between the HR of the mixed forest and the built environment insignificant (*F* (1, 55) = 0.90, *p* = 0.412; 95% CIs-coniferous forests = 73.03, 81.50; 95% CIs-broad-leaved forests = 74.93, 82.83; 95% CIs-mixed forests = 76.74, 86.73). And each 1-min average HR of MCB was the highest in these natural environments.

#### Heart rate variability

**Figure 4:**
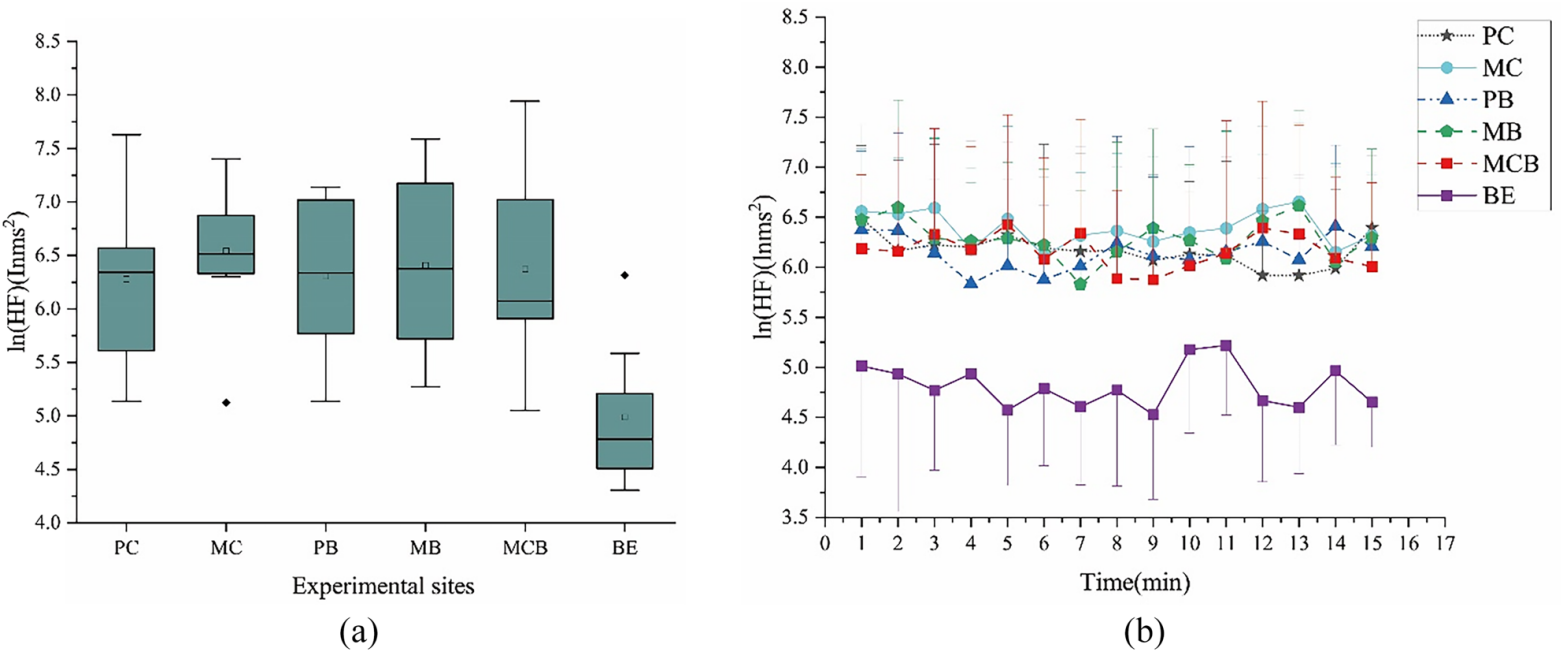
Mean 1-min ln(HF) and mean overall ln(HF) during viewing in natural and built environment. (**a**) Overall mean ln (HF); (**b**) Changes in each 1-min average ln (HF) over the 15-min watch; N = 57.

As shown in Fig. 4, during the 15-min exposure period, the subjects who spent time in the built environment had significantly lower ln (HF) (4.99 ± 0.60), than the subjects who were in the natural environment (6.38 ± 0.75) (*F* (1, 55) = 26.69, *p* < 0.001; 95% CIs-NE (natural environment) = 6.16, 6.60; 95% CIs-BE (built environment) = 4.50, 5.47). The ln (HF) measured in the participants who were in the plant communities had noticeable advantages, however, there was no significant differences among the groups. Figure 4b shows the fluctuations in ln (HF) of the participants in various environments during the 15-min exposure period. It can be seen that the average ln (HF) per minute observed in every natural environment was higher than in the built environment. However, the fluctuations of ln (HF) in each environment were obvious during the whole exposure period.

**Figure 5:**
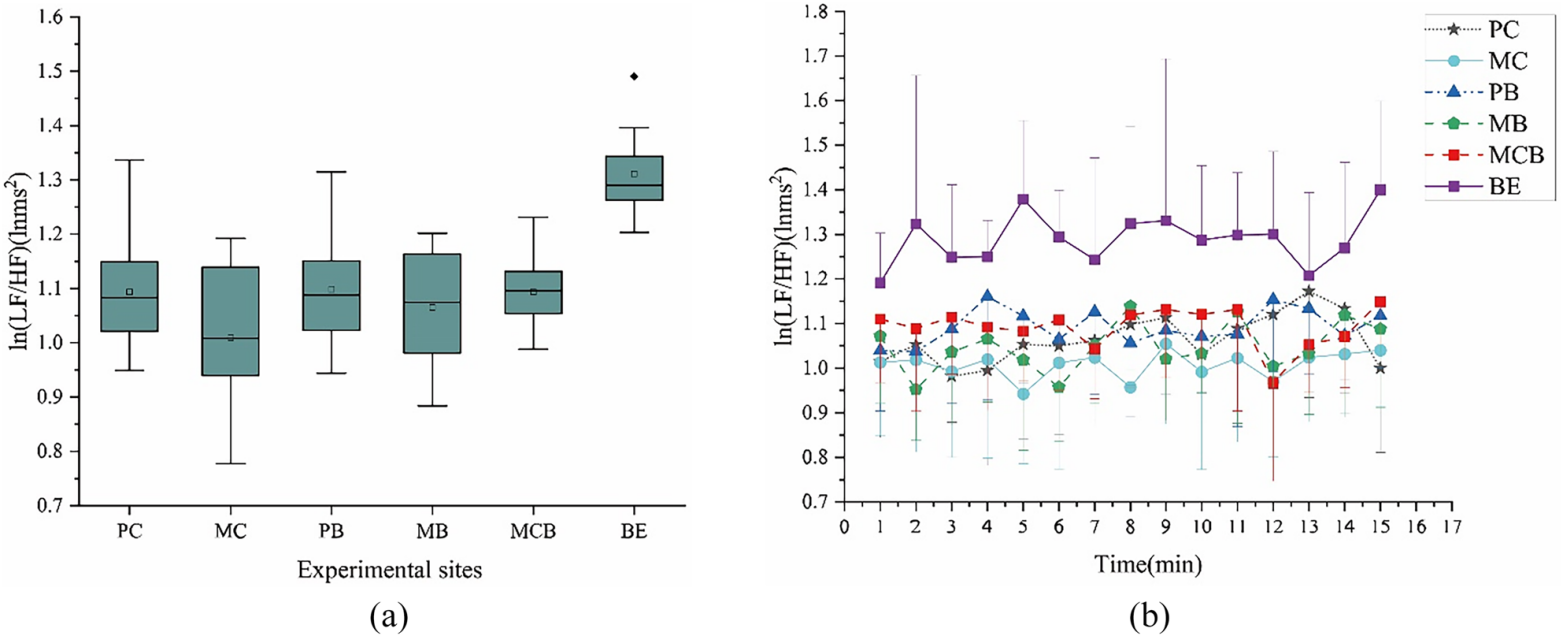
Mean 1-min ln(LF/HF) and mean overall ln(LF/HF) during viewing in natural and built environment. (**a**) Overall mean ln (LF/HF); (**b**) Changes in each 1-min average ln (LF/HF) over the 15-min watch; N = 57.

The natural logarithm of LF/HF is used to measure the relative activity of both sympathetic and parasympathetic nerves of the subjects. Lower values indicate lower levels of stress. Participants in the natural environments reported feeling more relaxed and calmer than those who were in the built environment. The lowest ln (LF/HF) value was observed in the participants who were in MC (1.035 ± 0.13), followed by those in MB (1.048 ± 0.10) (Fig. 5a). And ln (LF/HF) fluctuated greatly for all of the environments, but overall, the values observed in the built environment was always higher than the ones in the natural environments. Comparatively, the ln (LF/HF) variation of MC was relatively stable and always in a low state (Fig. 5b).

#### Salivary cortisol

**Figure 6:**
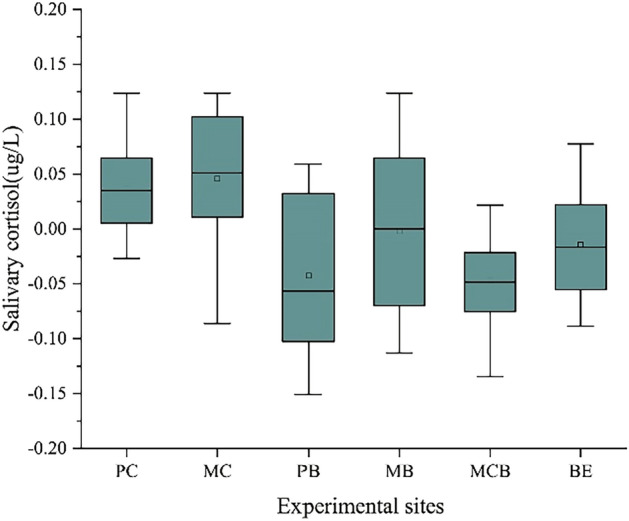
Changes in saliva cortisol concentration of the subjects; N = 59.

There was no significant change in the salivary cortisol concentration measured in the participants in both built and natural environments. After the experiment, the salivary cortisol concentration of those spending time in the natural environment was 2.278 ± 0.145 ug/L, while the subjects in the built environment had 1.869 ± 0.124 ug/L. However, the changes of salivary cortisol concentration in the three types of plant communities were different. And the environment which contributed to the largest decrease in the salivary cortisol was MCB (-0.047 ± 0.044 ug/L), followed by PB (-0.043 ± 0.071 ug/L). Compared with the changes of saliva cortisol concentration in broad-leaved forest and mixed forest, coniferous forest significantly increased saliva cortisol concentration (*F* (2, 46) = 7.516,* p* = 0.001 < 0.05; 95% CIs-coniferous forests = 0.015, 0.067; 95% CIs-broad-leaved forests = − 0.059, 0.015; 95% CIs-mixed forests = − 0.083, − 0.011) (Fig. 6).

### Psychological effects of natural environment on human stress

#### POMS

**Figure 7:**
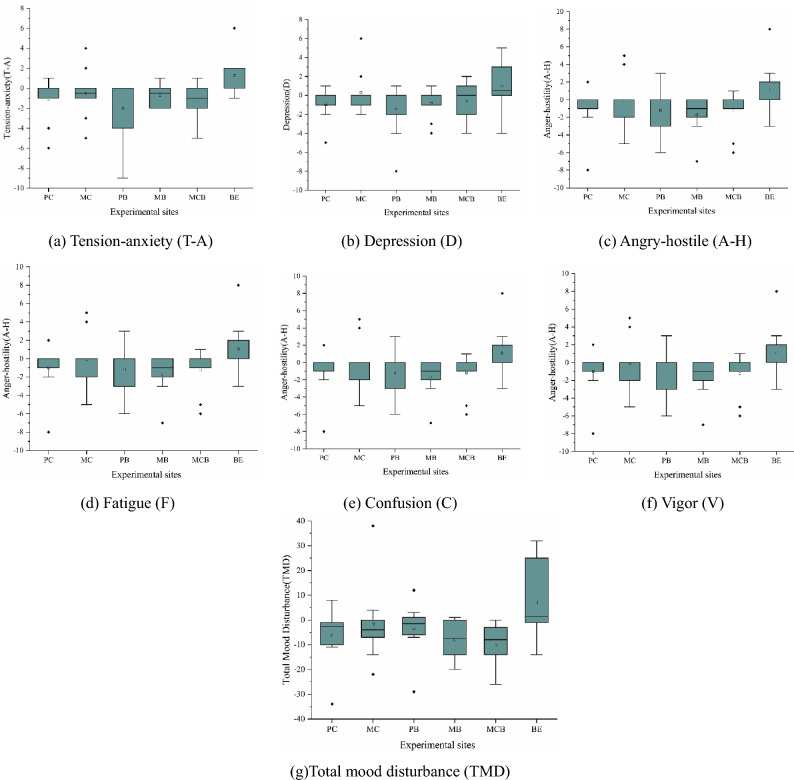
Changes of POMS subscales and TMD scores. (**a**) Tension-anxiety scale; (**b**) Depression scale; (**c**) Angry-hostile scale; (**d**) Fatigue scale; (**e**) Confusion scale; (**f**) Vigor scale; (**g**) Total mood disturbance; N = 59.

Figure 7 shows the changes of six POMS subscales and TMD scores of the participants after exposure to different environments. Compared with those who spent time in the built environment, the emotional state of subjects who were in the natural environment significantly changed after the exposure. In the five negative subscales, the negative emotions of the subjects who were in the natural environments substantially declined, while they significantly increased for those in the built environment. According to the results of the vigor subscale, the vitality status of the subjects in the natural environment improved, while it declined for those in the built environment. Furthermore, the change in TMD also confirmed the positive effect the natural environment had on the human emotions.

Except C and V subscale, Kruskal–Wallis test results of others scores of subjects in natural environment and built environment were significantly different (*p* < 0.05). In the V subscale, there were significant differences between broad-leaved forest and mixed forest, but there were no significant differences in other scores. More specifically, the participants in PB had the greatest decline in the T-A and D subscale (Fig. 7a,b). Furthermore, the lowest scores of T-A and D subscale were found in those who spent time in PC and MB. The score of the A-H subscale decreased most for the participants who were in MB (− 1.70 ± 2.00), while the F subscale score decreased most for those who spent time in MCB (− 3.33 ± 2.26). In particular, compared with the other two kinds of groups, the score of V subscale of the participants in the broad-leaved forest decreased slightly. When it comes to the TMD scores, the most substantial decline was noticed with the subjects who were exposed to MCB (− 10.22 ± 8.90). Although there was no significant difference in score changes in the other two subscales (C and F), most of the results confirmed the assumption that the natural environment can relieve the negative emotions and enhance the vitality (Fig. 7e,f).

#### STAI

**Figure 8:**
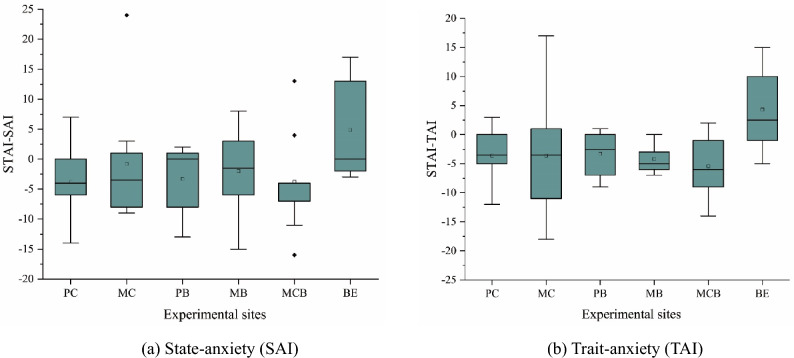
Changes of STAI score. (**a**) State-anxiety (SAI); (**b**) Trait-anxiety (TAI); N = 59.

As shown in Fig. 8, the anxiety level of the subjects was significantly lowered after the exposure to the five plant communities (SAI *p* = 0.013; TAI *p* = 0.001, which tested by Kruskal–Wallis), serving as the evidence of the relieving effect the natural environment has on human anxiety. On the other hand, the anxiety level of the subjects exposed to the built environment was intensified. In the SAI subscale, the lowest score after the exposure was noticed in the subjects who spent time in MB (31.60 ± 6.53), however, the greatest decrease was measured for the participants who were in the PC (-3.80 ± 5.53). When it comes to the TAI subscale, the scores of all of the subjects who spent time in nature dropped significantly. After the exposure, the highest TAI score was observed in those who spent time in the MC (38.20 ± 11.75), followed by MCB (35.67 ± 6.24), PC (35.50 ± 7.68), PB (35.40 ± 7.86), and MB (34.90 ± 7.24) (Fig. 8b). The results of multiple testing showed no significant difference between groups of different plant communities.

## Discussion

### Changes in stress state before and after exposure to natural environment

In recent years, numerous studies have reported the potential health benefits of natural exposure, confirming the deep connection between people and their natural environment^28,51,52^. The concept of “therapeutic landscape” also summarizes the enhancement of multi-dimensional happiness caused by natural environment, including human body, material, spirit and society^50^. In this study, a field experiment was conducted in both natural and built environments to investigate the impact of the natural environment on human stress reduction. Our results showed that compared with the built environment, the participants’ physiological and psychological stress levels were significantly reduced after the exposure to the natural environment. Participants exposed to the natural environment were calmer and more relaxed, while the negative emotions like depression and anxiety significantly reduced. Furthermore, their vitality increased, which was over all in line with previous research^47,50,53–55^.

In many Japanese studies, the HR and HRV were utilized to measure the function of the human autonomic nervous system in a short period of time^44,56^. As with other articles studying natural exposure and human health, subjects exposed to natural plant communities had higher HF, lower HR, and LF/HF ratio. This indicates that their parasympathetic nerve activity increased, improving their levels of relaxation and decreasing stress^47,57,58^. The changes in blood pressure and pulse rate were used to describe the cardiovascular health of the subjects. However, they were not significant, although there was a decrease in the pulse rates of those who spent time in the natural environment. Song et al.^44^ noticed that the subjects’ physiological responses may be related to the initial values of their measurement parameters. They observed that the subjects who initially had low blood pressure and pulse rate had these values increased after walking into a forest. This may be the explanation behind the insignificant results regarding blood pressure and pulse rate. Other studies have shown that some happy emotions can also increase blood pressure, which is inconsistent with reduced blood pressure during relaxation^59^. Similarly, we did not observe significant changes in salivary cortisol, which could be caused by many complex social factors, individual differences and circadian rhythms, etc*.*^60^. Previous studies have also observed the same results^61,62^.

Viewing the landscape of a plant community increased the participants’ energy levels and eased negative emotions such as depression, fatigue, anxiety, and confusion, according to the psychological evaluations. Additionally, the participants’ TMD, TAI, and SAI levels all decreased significantly, which is in line with the previous findings^16,27,52,63^. In recent years, the role of natural exposure in improving spirit and resilience has been verified, and people also call for natural immersion as an intervention means of mental health^64–67^. Mental and emotional health is a driver and exacerbator of social inequality^68^. Urban green space, which has an important impact on human emotions, has also received greater attention in public policies and public discourse.

### Effects of different plant communities on human stress state

In addition, it was discovered that plant communities with varying compositions had different effects on human stress levels. When all physiological indicators were considered, MC had the most positive effect on reducing the subjects’ physiological stress when compared to the rest of the plant communities studied. It was composed of some coniferous plants, such as *Juniperus formosana*, *Pinus bungeana*, *Cedrus deodara*, and others. These plants were commonly used in rehabilitation landscape, and they could release volatile components such as olefin compounds, terpenoids, alcohols, which were beneficial to human health^35,69^. Studies have also proved that these conifers can purify the air, which also briefly affects human health^70,71^. Another plant community that reduced physiological stress was composed of *Eucommia ulmoides* (main tree species of PB), which was shown to have anti-bacterial, anti-cancerous, cardioprotective, and neuroprotective properties^72^. *Eucommia ulmoides* had great benefits to human health, which made some physiological indexes of subjects exposed to *Eucommia ulmoides* forest have very positive changes. These changes meant the reduction of human physiological stress. Both An et al.^73^ and Elsadek^31^ studies examined the intricacies of human health resulting from the microenvironmental differences caused by various plants, but our findings indicate that the plant characteristics and properties may also have varying effects on human health. In this situation, some frequently-used plants in rehabilitation landscape or the combination of them may directly bring benefits to human health.

Similarly, only broad-leaved forest and mixed forest scored significant differences in the V subscale. Some previous studies on the health effects of parks, woodlands, or forests with different degrees of wilderness also showed that there was no significant difference in the mental health based on the environment^74,75^. Among the plant communities in this research, MCB had the best effect on relieving the psychological stress of subjects, as well as their mood and anxiety levels. Therefore, it was speculated that the high number of plants released volatile components (such as *Cedrus deod*ara, *Metasequoia glyptostroboide*s and *Robinia pseudoacacia*), while the tidier and transparent arrangement of plants in this community had more positive effects on human psychological stress. This result was in line with the research by Takayama^32^ and Gatersleben and Andrews^76^. They noticed that a more organized environment, with clear fields of vision (prospect) and few hiding places (refuge) had better recovery. Bratman et al.^77^ divided the approach of environment and mental health into four steps, among which the first step is to describe and define “natural features”. This step states that the size, composition and spatial configuration of natural landscapes, as well as other natural attributes (vegetation features, structure and biodiversity, etc.) may be natural element characteristics that influence mental health. This is also consistent with our findings.

At the moment, there is some debate as to whether mixed forests are the best type of forests for recreation, but plant diversity can have a direct or indirect impact on the potential of green space^51,78^. Our study showed that mixed coniferous and broad-leaved forests were more effective than single coniferous forest or broad-leaved forest in relieving physical and psychological stress. The reason for this difference might be that various plants affected the external morphology and spatial structure of the plant community, or that these plants produced more oxygen or beneficial volatile substances, and other potential influencing factors. Some studies say that the characteristics of natural landscape will affect a series of reactions such as "exposure-experience-effect" of human body, not to mention the influence of natural experience will also be affected by the age, gender, emotional state and natural preference of the subjects^77^. This relationship needs to be further examined and discussed in future studies.

### Limitations and future development

This research studied the effects of various typical plant communities on human stress relief. For the purpose of the research, the plant communities were screened for five different components, which was not often seen in the previous studies. However, the research still had some limitations. A total of 59 volunteers participated in the experiment, and they were divided into six groups of 10. There were few subjects in each group to control for the personal characteristics of the subjects. In addition, since gender ratio of volunteers was imbalanced and the subjects in each sample site were randomly assigned, the influence of gender on the results cannot be excluded. Although the experimental area was strictly controlled, the tourists visiting the Badaguan Scenic Area and the surrounding locations could have influenced the subjects. Besides, the cultural values of the subjects about the natural environment are different. For example, simple preferences for conifers and broad-leaved forests may vary from country to country.

Based on the above limitations, to further explain the differences in the effects of the different plant communities on human stress levels, a more comprehensive study needs to be conducted. Particularly, the question remains whether the result could be repeated among people of different ages and different health levels. Future research should include more diverse experimental groups, such as elderly people with impaired physical health or people with certain physiological or psychological disorders. Another important issue is that these plant communities in Badaguan Scenic Area are disturbed artificially. Whether the plant communities occurring in a more natural environment have the same effect on human stress levels are still an unanswered question. Moreover, the effects of the natural environment on health are not limited to static interactions with the natural environment, some physical activities and social interactions could also be included in the future experiments.

## Conclusion

In the present study, we attempted to elucidate the role of the natural environment on human stress relief and to compare the diverse effects of different typical plant communities on human stress reduction. Therefore, the typical plant communities in Badaguan Scenic Area were selected and a field experiment was conducted. The results showed that compared to the built environment, the natural environment can relieve both physiological and psychological stress and the negative emotions while significantly increasing vitality. Different plant communities also have different effects on human health, for example, MC can better relieve the physiological stress of the subjects, and MCB has a significant effect on improving emotional state and reducing anxiety. Broad-leaved forest, mixed forest and coniferous forest significantly affected DBP, activity level and salivary cortisol concentration, respectively. The study confirmed the stress-relieving effect of small-scale plant communities and highlighted their health benefits. Based on this, the application of various plant species and the combing of plant community space may become the foothold and breakthrough of healthy city construction. Renewal and improvement of plant community spatial structure and plant allocation in urban green space should also become an important means to build a healthy and livable city. On the other hand, the research on the relationship between the composition and spatial structure of the small-scale plant communities and human health is limited. More extensive research and repeated experiments are required to provide a more scientific basis for plant allocation and the creation of healthy urban green spaces.

## Data Availability

The original contributions presented in the study are included in the article, further inquiries can be directed to the corresponding author.
